# Dynamic MRI of the vocal cords using phased-array coils: A feasibility study

**DOI:** 10.4103/0971-3026.50830

**Published:** 2009-05

**Authors:** Marc Schlamann, Götz Lehnerdt, Stefan Maderwald, Susanne Ladd

**Affiliations:** Department of Diagnostic and Interventional Radiology and Neuroradiology, University of Duisburg-Essen Medical School, Essen, Germany; 1 Department of Otorhinolaryngology, University of Duisburg-Essen Medical School, Essen, Germany

**Keywords:** Dynamic MRI, phased-array coils, vocal cords

## Abstract

**Objective:**

Endoscopy for evaluation of hoarseness is an invasive procedure and the result depends, to a large extent, on the patient's cooperation. Successful laryngoscopy can also be hampered by unfavourable anatomic conditions, a severely impaired general condition, or severe coagulopathy. We evaluated the feasibility of doing ultra-fast magnetic resonance imaging (MRI), using a recent dedicated coil design and a sequence with inherently high signal-to-noise ratios (SNR), for the detection of motility disorders of the vocal cords.

**Materials and Methods:**

Twelve consecutive patients (eight males and four females) in the age range of 24–80 years (mean age 60 years) with persistent hoarseness and presumed vocal cord palsy were included in this blinded prospective study. Two two-element phased-array carotid coils were used for signal reception. The first coronal real-time steady-state free precession (SSFP) sequence was performed during silence (i.e., with no vocal cord motion) and the second while phonating ‘heee.’ Qualitative MRI findings were compared with the results of the endoscopic examination.

**Results:**

The examination time for setup, patient instruction and positioning, localization scans, and real-time SSFP scans was less than 10 min. Seven patients with laryngoscopically-confirmed unilateral palsy of the vocal cord were correctly identified with MRI. The five remaining patients had hoarseness due to causes other than vocal cord palsy; they showed normal motion of the vocal cords on MRI and endoscopy.

**Conclusion:**

Compared to preceding studies, the image quality in this study is supported by excellent SNR (carotid phased-array coils and SSFP sequence with higher SNR if compared to a spoiled gradient-echo sequence or an EPI sequence). Further studies, with larger groups of patients, are necessary to show if this protocol can serve as an alternative to endoscopy in selected cases.

Hoarseness is a common symptom in a variety of benign diseases; for example, it may be due to infection and inflammation (e.g., common cold), vocal cord paralysis, or trauma[1]. However, this symptom may also be due to malignant tumours. Müller[2] proposed that all cases of hoarseness lasting longer than 3 weeks must be evaluated by an otorhinolaryngologist to rule out malignant disease.

Although modern endoscopic devices are extremely flexible, the examination is nevertheless invasive; successful endoscopy depends to a large extent on the patient's cooperation. Moreover, the procedure might be impossible to perform in some cases due to unfavourable anatomic conditions, abnormal emetic disposition, severely impaired general condition of the patient, or severe coagulopathy.[3]

Radiological fluoroscopic imaging has been used for decades as an alternative to endoscopy. While fluoroscopy provides real-time images of the vocal cords during different phonations, it does not display the surrounding anatomy. The search for underlying pathologies must then be performed by means of other techniques. In cases of vocal cord palsy USG is often used to look for underlying causes such as tumors. In adults, however, specific examination of the vocal cords using USG may be hampered by echoes caused by laryngeal calcifications and air–tissue borders.[4]

CT scan is usually used for assessing tumor extent or for the presence of anatomic variants of vessels or nerve courses and can also be used for virtual laryngoscopy.[5,6] Studies have been conducted that have used sequential scans during different phonations.[7] Some of these studies have shown that coronal reconstruction of images acquired during ‘hee’ phonation could improve the detection rate of vocal cord palsy.[8]

Like CT scan, MRI enables the examiner to visualize pathological changes beneath the surface of the larynx and, for example, discover a tumor in the soft tissue or estimate the depth of invasion of a malignant process. Theoretically, at least, MRI provides better tissue contrast as well as sufficient spatial resolution for visualization of the vocal cords. The main advantages of MRI in this context, however, are that it allows real-time dynamic imaging of moving structures with adequate temporal resolution and also allows the radiologist to choose adequate imaging planes beyond the axial orientation.

A small series of MRI studies has been published on the potential of this modality in the assessment of the vocal tract.[9–15] In addition to triggered MR techniques, which are all quite laborious, few publications exist which use ‘real-time’ imaging, mainly using spoiled gradient-echo sequences or echo-planar-imaging.[12] These authors were able to detect vocal cord paralysis in all six patients with EPI sequences but had to use an additional coating filled with chlorinated fluorocarbon to maximize field homogeneity. Hirayama *et al*.[9] used turbo spin-echo sequences in a low-field scanner and achieved a temporal resolution of 0.9 s. Modern ultra-fast sequences combined with small phased-array surface coils, such as are used for carotid imaging, offer very high signal-to-noise ratios (SNR) and thus theoretically allow for even faster dynamic imaging, with higher spatial and temporal resolution.[13] Using these recent technological improvements, we evaluated the feasibility of ultra-fast MRI in the detection of motility disorders of the vocal cords.

## Materials and Methods

Twelve consecutive patients (8 male and 4 female; age range 24–80 years; mean age 60 years) with persistent hoarseness of more than 2 weeks' duration were included in this prospective study over a 3-month period. After initial clinical examinations in the ENT department, patients with presumed vocal cord palsy had MRI done. All MRI exams were performed using a 1.5-Tesla scanner (Magnetom Sonata, Siemens Medical Solutions, Erlangen, Germany) with high-performance gradients (40 mT/m maximum amplitude, 200 mT/m/ms slew rate). To achieve higher SNR in comparison to the standard coils (neck coil), two two-element phased-array carotid coils (Machnet BV, Eelde, The Netherlands) were used for signal reception. Each coil covers an area of 105 × 60 mm and has a penetration depth of approximately 35 mm.

**Figure 1:**
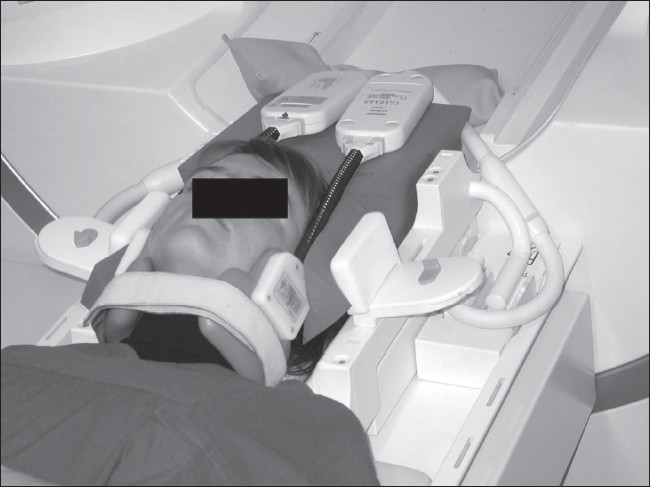
Setup with Machnet carotid coils supported on an unplugged head coil with sandbags

The patients were positioned head first in the supine position on the scanner table and the two carotid coils were placed on either side of the neck. The base of the unplugged head coil and small sandbags were used for fixation [Figure 1]. For determination of the exact location of the vocal cords, the MRI protocol started with fast (turbo) spin-echo scout scans in all three orientations. Subsequently, two adjacent coronal images were repetitively acquired with a shared-phases real-time SSFP (steady-state free precession sequence with balanced gradients; TR: 282 ms, TE: 1.3 ms, flip: 55°, FOV: 235 × 117.5 mm^2^, matrix: 128 × 128, resulting pixel size: 1.8 × 0.9 mm^2^, slice thickness: 4 mm) with a temporal resolution of 4 images per second over 14 s (56 images). The first (baseline) real-time SSFP was performed during silence (no vocal cord motion), the second while phonating the sound ‘heee’ (which is used in otorhinolaryngological examination for prompting movement of the vocal cords).

The MRI scans were analyzed by two experienced radiologists in consensus, with respect to imaging quality, presence or absence of palsy, and possible causes for the palsy. The readers were blinded to the clinical results as far as possible. Palsy of a vocal cord was assumed if one or both vocal cords showed no movement during phonation.

After the MRI scan, all patients were examined laryngoscopically to identify possible paralysis of the vocal cords. The examiner, from the department of otorhinolaryngology, was blinded to the results of the MRI scan. Finally, the MRI findings with regard to the movement of the vocal cords were compared to the results of the endoscopic examination, which is still considered the ‘gold standard’ for evaluation of diseases of the vocal cords. Any additional information obtained either by the dynamic sequence or by the contrast-enhanced morphological sequence was recorded.

## Results

**Figure 2:**
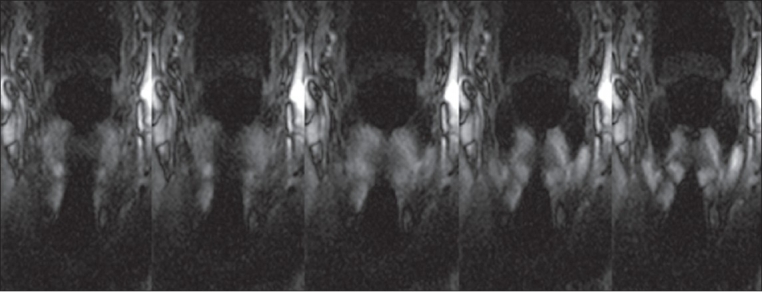
Patient XII with postoperative hoarseness: Five time points of the second coronal SSFP sequence while phonating ‘heee.’ The symmetric movement of the vocal cords is clearly visible

**Figure 3:**
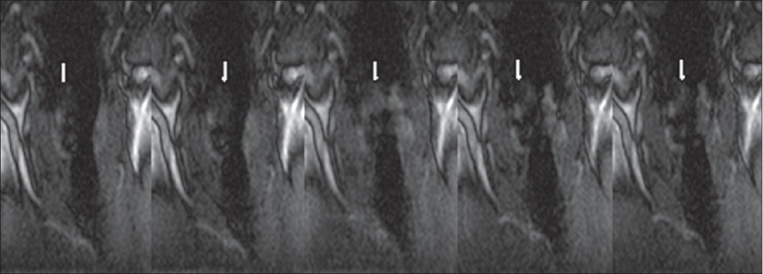
Patient I; the unilateral palsy of the right vocal cord (arrows) can be seen in the displayed 5 of 56 time points

The examination time of less than 10 min for setup, patient instruction and positioning, localization scans, and real-time SSFP scans, was well tolerated and the examination was successfully completed by all 12 patients. Normal vocal cord movement or palsy could be correctly identified in all cases. Table 1 shows the results of the 12 patients. Seven patients had laryngoscopically-confirmed unilateral palsy of the vocal cord. These patients were correctly identified by dynamic MRI. Figure 2 illustrates a palsy of the right vocal cord and normal movement of the left vocal cord, while Figure 3 shows the normal situation. The five remaining patients had hoarseness due to causes not related to palsy of the vocal cords, e.g., leukoplakia of the vocal cords, glottic cancer, papillomatosis, and postoperative hoarseness; they showed normal motion of the vocal cords on MRI and endoscopy. In one case (patient VII in Table 1) the exact reason for the hoarseness could not be identified either by MRI or by clinical examination.

**Table 1:** Clinical data of all 12 patients

Patient	Age	Sex	Vocal cord palsy	Laryngo-scopy	MRI	Cause	Disease/other reason for hoarseness
I	24	F	Right	Y	Y	Recurrent laryngeal nerve (RLN) palsy	Mediastinal manifestation of M. Hodgkin
II	65	M	Left	Y	Y	Luxation of the arytenoid cartilage	Post intubation
III	69	M	None	N	N		T1a N0 M0 glottic cancer
IV	46	M	None	N	N		Leukoplakia
V	56	M	Right	Y	Y	RLN palsy	
VI	62	M	Left	Y	Y		Lung cancer
VII	75	M	None	N	N		Unknown
VIII	80	F	None	N	N		Papillomatosis
IX	73	F	Right	Y	Y	Vagal nerve palsy	
X	36	M	Left	Y	Y	RLN palsy	Aortic dissection
XI	61	M	Right	Y	Y	RLN palsy	Sarcoma
XII	75	F	None	N	N		Postoperative hoarseness

## Discussion

This study was performed to assess the feasibility of a combination of a dedicated coil and a high signal-rendering SSFP sequence in the diagnosis of vocal cord palsy. Dynamic real-time MRI was able to reliably detect the presence of vocal cord palsy and identify the side involved in all 12 patients. Furthermore, no false positive diagnosis occurred.

Although not assessed in detail, the setup of this examination is easy compared to that in other studies. Two adjacent real-time images can be acquired with a temporal resolution of 4/s, the image quality as provided by the carotid coil and the SSFP sequence is better compared to EPI or spoiled gradient-echo sequences,[16,17] and the total examination duration is as low as 10 min. Artefacts due to susceptibility effects at air–tissue borders are marginal.

**Figure 4:**
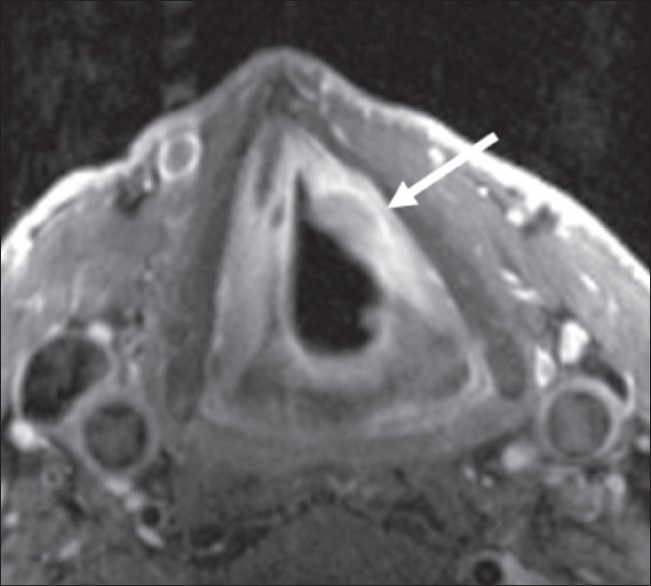
T1a carcinoma of the left vocal cord (arrow). Expansion and penetration depth are clearly visible

The application of contrast media is not necessary if only motion is to be assessed. However, in case a pathologic structure is seen in the localizers or the dynamic images, or if there is clinical suspicion of malignant disease, subsequent contrast media application might improve the diagnostic efficiency [Figure 4]. Although we have not compared the neck volume coil with the carotid coils that were used in this study, we presume that the addition of the dynamic sequence to a routine tumor protocol would require repositioning of coils due to a restricted field-of-view in the z-direction of the carotid coil.

Because of its high cost as compared to conventional laryngoscopy, MRI certainly cannot be considered a routine screening tool for vocal cord paralysis and will not replace endoscopic techniques. However, in selected cases, where conventional laryngoscopy is not applicable, MRI might be a reasonable alternative. Further larger studies are needed to show if this protocol might serve as an alternative to endoscopy in selected cases.
